# Comparative 3‐Year Allograft Outcomes for Recipients of Kidneys From SARS‐CoV‐2 NAT‐Positive Donors

**DOI:** 10.1111/tid.70202

**Published:** 2026-03-13

**Authors:** Christine E. Koval, Mohamed Eltemamy, Nabin K. Shrestha, Leal Herlitz, Emilio D. Poggio, Alvin C. Wee

**Affiliations:** ^1^ Department of Infectious Diseases Respiratory Institute Cleveland Clinic Cleveland Ohio USA; ^2^ Department of Urology Glickman Urological and Kidney Institute Cleveland Clinic Cleveland Ohio USA; ^3^ Department of Pathology Institute of Pathology and Laboratory Medicine Cleveland Clinic Cleveland Ohio USA; ^4^ Department of Kidney Medicine Glickman Urological and Kidney Institute Cleveland Clinic Cleveland Ohio USA

**Keywords:** kidney transplantation, SARS‐CoV‐2

## Abstract

**Background:**

Data demonstrates safety and short‐term success of kidney transplantation (KT) from SARS‐CoV‐2 NAT‐positive organ donors (NAT+) compared to NAT‐negative donors. However, detailed reports of longer‐term graft outcomes are limited and lack granular data on donor SARS‐CoV‐2 infection severity.

**Methods:**

We retrospectively reviewed records from 220 recipients of KT (KTRs) at our institution from February 1, 2021, to January 31, 2022. We compared 1‐ and 3‐year allograft‐specific outcomes for KTRs from NAT+ (*N* = 59), CoV‐COD (COVID as cause of death) (*N* = 56), and NAT‐neg (*N* = 105) donors. We reviewed and compared pathology findings for allograft biopsies for all groups up to 1 year. We examined the association of eGFR at 3 years with CoV‐donor status using multivariable linear regression.

**Results:**

At 1 year, there was no difference in rejection or kidney pathologies by CoV‐donor status. At 3 years, there was no difference in patient death or graft loss by group. For subjects without graft loss, eGFR at 3 years for NAT+, CoV‐COD, and NAT‐neg donors was 54.8 (21.8), 45.6(17.9), and 48.4 (23.3) mL/min/m^2^, respectively (*p* = 0.61). On multivariable analysis, there was no significant association of eGFR with CoV‐donor status.

**Conclusions:**

We found no significant difference in pathological findings or 3‐year allograft outcomes following KT from NAT‐negative, NAT+, or CoV‐COD donors. Despite modestly reduced eGFR for COV‐COD on unadjusted analysis, kidneys from donors with a spectrum of COVID severities did not experience significantly poorer allograft outcomes over 3 years.

## Graphical abstract

In this retrospective single‐center study of 220 kidney transplants from SARS‐CoV‐2 NAT+, NAT+ with COVID as cause of death (CoV‐COD) and NATneg donors, we found no significant difference in rejection or kidney pathologies at 1 year, and death, graft loss, or eGFR at 3 years by CoV‐donor status.

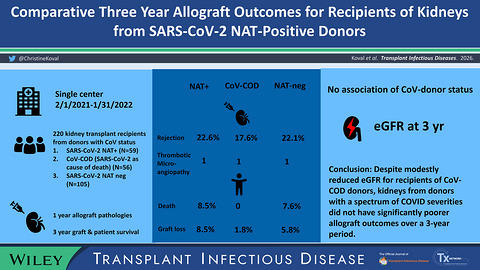

**Abbreviations**
ACR: acute cellular rejection
AMR: antibody‐mediated rejection
CIT: cold ischemia time
CoV‐COD: COVID cause of death
COVID‐19: coronavirus disease 2019
DBD: donation after brainstem death
DCD: donation after cardiac death
DSA: donor‐specific antibodies
eGFR: estimated glomerular filtration rate
KDPI: kidney donor profile index
KT: kidney transplant
KTR: kidney transplant recipient
NAT: nucleic acid test
rATG: rabbit thymocyte globulin
SARS‐CoV‐2: severe acute respiratory syndrome coronavirus 2
TMA: thrombotic microangiopathy
UNOS: United Network for Organ Sharing

## 1 Introduction

The general safety of extra‐pulmonary organ transplantation from SARS‐CoV‐2 nucleic acid test‐positive (NAT+) donors has now been well established [1, 2, 3, 4, 5]. Despite plausible mechanisms, concerns for transmissible infection from hearts, kidneys, and livers appear to be unfounded, with no single case of proven SARS‐CoV‐2 transmission occurring from any organ other than lungs [6, 7, 8, 9, 10, 11].

Despite overall acceptance of transplantation from SARS‐CoV‐2 NAT+ donors, some concerns remain regarding the subclinical effects of active or severe COVID‐19 on extrapulmonary organs and the need for closer scrutiny of organs from such donors. COVID‐19 can result in acute kidney injury and renal failure [12, 13, 14]. Autopsy reports from COVID‐related deaths and live biopsies from those with AKI identified characteristic pathologic effects, including thrombotic microangiopathy (TMA), collapsing glomerulopathy, and diffuse tubular injury [15, 16]. However, longer term effects of less severe cases of COVID on renal function may be of minimal consequence [17].

We previously reported very good patient and allograft outcomes for over 100 kidney transplants from SARS‐CoV‐2 NAT+ donors (with and without COVID as the cause of death) compared to those from SARS‐CoV‐2 NAT‐negative donors with a median follow‐up of 5.7 months [1]. We aimed to describe 3‐year comparative outcomes for these same recipients with a focus on allograft function and pathological findings.

## 2 Methods

This is a retrospective study of all single adult kidney transplant recipients of deceased donors performed at the Cleveland Clinic from February 1, 2021, to January 31, 2022. We collected deceased donor data, including details of SARS‐CoV‐2 testing and infection, and recipient clinical and laboratory baseline and follow‐up data. Donors were NAT+ if SARS‐CoV‐2 RNA was detected within 72 h of procurement, with no evidence for recently resolved infection. Donors were CoV‐COD if they died of hypoxic respiratory failure due to SARS‐CoV‐2 with SARS‐CoV2 RNA detected within the index hospitalization. Recently resolved infection was defined as a history of confirmed COVID‐19 with the resolution of symptoms and signs 21–90 days from the date of symptoms onset. This retrospective study was approved by the Cleveland Clinic Institutional Review Board as an exempt study not requiring patient consent for investigation or publication. There is no clinical study registration number.

As previously described, our protocols for considering SARS‐CoV‐2 NAT+ donors evolved through 2021 [1]. There were no recipient selection criteria other than the willingness to accept and consent to the use of such an organ. By November 2021, all adult transplant candidates at our institution were required to be fully vaccinated with at least two doses of an mRNA vaccine or one dose of SARS‐CoV‐2 viral vector vaccine (Johnson and Johnson's Jannsen) as a condition for transplantation. During the study period and prior to transplant, 88%, 93.2%, and 100% of recipients of NATneg, NAT+, and CoV‐COD donors, respectively, were either vaccinated or had prior resolved COVID. All recipients received standard immunosuppression, typically including induction with rabbit anti‐thymocyte globulin (rATG), tacrolimus, mycophenolate mofetil, and prednisone. Recipients received no pre‐emptive anti‐SARS‐CoV‐2 therapies, including direct‐acting agents or passive antibody therapies. Recipients were monitored for signs of SARS‐CoV‐2 clinical infection and standard markers of allograft function.

Post‐transplant creatinine and eGFR were determined within 2 months around the 3‐year time from transplant. eGFR was calculated using the 2021 CKD‐EPI 2021 creatinine equation based on serum creatinine, age, and sex as parameters.

“Per protocol,” biopsies were obtained at 3–6 and at 12 months unless contraindicated. “For cause” biopsies were obtained in the setting of otherwise unexplained allograft dysfunction and reported for the first transplant year. Allograft rejection was defined by biopsy as proven acute cellular rejection (ACR), borderline ACR (bACR), antibody‐mediated rejection (AMR), or “mixed” (ACR + AMR) according to the Banff criteria (https://banfffoundation.org/) that led to directed therapies for these diagnoses. TMA was pathologically descriptive and defined in the absence of active allograft rejection.

### 2.1 Statistical Analysis

We previously compared demographic and clinical characteristics of donor organs based on NAT+, CoV+COD, and NATneg status, as well as recipient characteristics based on receipt of these organs. We have included key comparative differences identified from our earlier analysis. We used chi‐square and Fisher's exact tests to compare the associations of categorical characteristics and one‐way analysis of variance for continuous variables as appropriate. We used multivariable linear regression to compare the 3‐year estimated glomerular filtration rate (eGFR) between recipients of NAT+, CoV+COD, and NAT‐neg kidneys. We adjusted for covariates that were identified as potentially associated with follow‐up eGFR, which included the kidney donor profile index (KDPI), cold ischemia time, recipient age at transplantation, gender, body mass index (BMI), and either donation after cardiac death (DCD) or donation after brain death (DBD) donor.

## 3 Results

As described in Table 1, NAT+ donors were younger, less likely to be white, and more likely to be from outside of Ohio than NAT‐neg donors. CoV‐COD donors had higher BMI, were universally DCD, had longer cold ischemia times, and were also more likely to be from outside Ohio than NAT‐neg donors. As previously reported, recipients of NAT+ and CoV‐COD donors were more likely to be pre‐emptive and have shorter dialysis times than recipients of NAT‐neg donors, but were otherwise similar. There were 57 donors (46 NAT+ and 11 NAT‐neg) who provided both kidneys to separate recipients at our center.

**TABLE 1:** Donor characteristics by SARS‐CoV‐2 status.

	NAT+ donors *N* = 35	CoV‐COD donors *N* = 33	NAT‐neg donors *N* = 93	*p*‐value
Age, yrs, mean, sd	32 (12.1)	40.4 (12.1)	39.9 (13.2)	< 0.01
Mean KDPI, sd	36.1 (22.1)	40.6 (22.3)	45.5 (24.7)	0.12
DCD	12 (40%)	33 (100%)	43 (46.2%)	< 0.01
BMI kg/m^2^	27.9 (8.7)	38.5 (10.4)	30.5 (7.7)	< 0.01
Race/ethnicity, White	20 (57%)	28 (85%)	76 (82%)	0.02
CIT, hr, mean, sd	22.9 (6.3)	25.2 (6.6)	22.2 (6.7)	0.02
OPO Ohio	7 (20%)	2 (6%)	56 (60%)	< 0.01
VV ECMO	0	6 (18%)	1 (1%)	
Imaging, viral pneumonia	12 (34%)	33 (100%)		
SARS‐CoV‐2 RNA +, < 72 h	35 (100%)	31 (94%)		
Cycle threshold, mean, *N* = 26	28 (9.9)	31.9 (5.1)		
10–24	6 (33%)	1 (13%)		
25–34	6 (33%)	5 (62%)		
35–41	6 (33%)	2 (25%)		
Time from first + NAT result to donation, days, median (range)	3 (0–44)	27 (10–66)		

Abbreviations: BMI, body mass index; CIT, cold ischemia time; DCD, donation after cardiac death; KDPI, kidney donor profile index; OPO, organ procurement organization; VV ECMO, veno‐venous extracorporeal membrane oxygenation.

### 3.1 Three‐Year Patient and Graft Survival NAT+ and NATneg Donors

There were no significant differences in key post‐transplant outcomes for recipients of NAT+ or CoV+COD compared to NAT‐neg donors, including patient death and allograft loss (Table 2). Five of 59 recipients of KT from NAT+ donors died. Recipients of the mate kidneys from the same donors also performed at our center are alive with preserved allograft function. Eight of 105 KTRs from NAT‐neg donors died, including two from complications of severe COVID‐19 in the first‐year post‐transplant. Graft loss occurred in six recipients of NATneg donors, one recipient of CoV‐COD donors, and five recipients of KT from NAT+ donors who survived to 3 years. Recipients of the mate kidneys from the same NAT+ donors have preserved graft function.

**TABLE 2:** Comparing 1‐ and 3‐year kidney transplant recipient (KTR) outcomes based on donor type.

	KTRs NAT+ Donors *N* = 59	KTRs CoV‐COD Donors *N* = 56	KTRs NAT‐neg Donors *N* = 105	*p*‐value
Death, 3‐year	5 (8.5%)	0	8 (7.6%)	0.06
Graft loss, 3‐year	5 (8.5%)	1 (1.8%)	6 (5.8%)	0.30
eGFR, 1‐year, mL/min/m^2^, sd	56.53(19.57)	43.35 (18.51)	50.02 (21.07)	0.003
eGFR, 3‐year, mL/min/m^2^, sd	54.82 (21.84)	45.55 (17.89)	48.43 (23.28)	0.61
Rejection, 1‐year	12/53 (22.6%)	9/51 (17.6%)	17/77 (22.1%)	0.72
bACR	5	7	5	0.27
ACR	5	2	5	0.48
AMR	1	0	3	0.49
Mixed	1	0	4	0.33
Thrombotic microangiopathy	1	1	1	1.00

Abbreviations: ACR, acute cellular rejection; AMR, antibody‐mediated rejection; bACR, borderline ACR; eGFR, estimated glomerular filtration rate.

### 3.2 Pathologic Findings in Year 1

Seventy‐seven out of 105 (73%) NAT‐neg recipients had 111 biopsies in the first post‐transplant year. Of these 21 (18.9%) were “for cause” or for follow‐up of prior abnormality, with the remainder performed “per protocol.” Fifty‐three of 59 (89.8%) NAT+ donors had 75 biopsies, 12/75 (16%) “for cause.” Fifty‐one of 56 (91%) recipients of CoV‐COD donors had 69 biopsies with 12/69 (17%) “for cause.” Rejection resulting in treatment was identified in 22.6%, 17.5%, and 22% of recipients of NAT+, CoV‐COD, and NAT‐neg donors that underwent biopsy, respectively. Most cases were bACR (Table 2). There were no differences between groups in either AMR or ACR.

There were three biopsies (one in each group) with findings described as TMA (Table S1). One recipient of a kidney from a CoV‐COD donor after 4 years of peritoneal dialysis had a “for cause” biopsy performed 10 days after transplant in the setting of poor urine output and de novo donor‐specific antibodies (DSA), was treated with intravenous immune globulin (IVIG), eculizumab, and belatacept. A follow‐up biopsy demonstrated moderate tubular atrophy, interstitial fibrosis, and acute on chronic TMA. Creatinine at 18 months was 2.2 mg/dL, and urine demonstrated trace proteinuria. The mate kidney was not accepted at our center. One recipient of a kidney from a NAT+ donor had Sjogren's interstitial nephritis and pre‐transplant TMA that recurred 3 months post‐transplant in the setting of DSA and serum creatinine of 2.6. This was treated with eculizumab. This patient died of progressive interstitial lung disease (see above). The donor was a 6‐year‐old with brain death, KDPI of 59, a positive SARS‐CoV‐2 RNA test on Days 3 and 0 prior to donation, a normal chest x‐ray, and no clear signs of COVID‐19. The recipient of the mate's kidney had ACR detected at Month 6 and was treated with methylprednisolone. A third patient with a kidney from a NAT‐neg donor had TMA identified on biopsy following treatment for ACR at 6 months post‐transplant. This was not additionally treated. Creatinine at 18 months was 2.5 mg/dL. The recipient of the mate kidney had no pathologic findings on protocol biopsies.

### 3.3 Graft Function

Graft function, by eGFR values at 1 and 3 years are reported for all donor types in Table 2. On multivariable analysis (Table 3), higher recipient age at transplant, deceased donor status, and higher KDPI were associated with lower eGFR at last follow up. CoV‐donor status was not.

**TABLE 3:** Estimates of slopes from the regression model predicting estimated GFR at 3 years.

Variable	Point estimate (95% confidence interval)	*p*‐value
NAT+ donor	1.97 (−4.89, 8.83)	0.57
CoV‐COD donor	−1.50 (−8.97, 5.97)	0.69
Recipient age at transplant	−0.27 (−0.52, −0.01)	0.04
Recipient gender female	−1.22 (−6.81, 4.37)	0.67
Recipient BMI	−0.35 (−0.80, 0.10)	0.13
KDPI	−25.72 (−39.89, −11.55)	< 0.001
Deceased donor	−6.84 (−13.39, −0.28)	0.04
Cold ischemia time	−0.28 (−0.69, 0.13)	0.19

Abbreviations: BMI, body mass index; KDPI, Kidney Donor Profile Index.

## 4 Discussion

In this detailed report with 3‐year follow‐up, we found similarly good patient and allograft outcomes for recipients of kidneys from SARS‐CoV‐2 NAT+, CoV‐COD, and SARS‐CoV‐2 NAT‐negative donors. This is important since concerns for subclinical organ effects of active SARS‐CoV‐2 infection and in those dying of COVID ‐related respiratory failure have been posited for extrapulmonary organs, including kidneys. COVID‐19, particularly severe disease early in the pandemic, may cause acute kidney injury with pathologic findings of endothelial injury with TMA, collapsing glomerulopathy, and diffuse tubular injury [15, 16].

Our study describes and compares specific pathologies between groups for a contemporaneous period by CoV donor status. There were higher rates of biopsy for recipients of NAT+ and CoV‐COD kidneys, mostly for protocol purposes, indicating that these kidneys were well‐surveilled. We found no difference in pathologic findings between groups, including rates of ACR or AMR.

We found pathological evidence for TMA in three patients, one in each group. One recipient of NAT+ donor and one recipient of CoV‐COD donor had TMA with coincident DSA treated with eculizumab. One of these recipients had a history of pre‐transplant TMA. TMA can occur following kidney transplant, as recurrence or as de novo TMA. TMA is a group of diversified syndromes due to endothelial injury with pathologic findings of microangiopathy and small vessel thrombosis [18]. Those syndromes that involve complement and platelet activation include hemolytic uremic syndrome (HUS), atypical HUS, catastrophic anti‐phospholipid syndrome, and thrombotic thrombocytopenic purpura. Features may overlap with disseminated intravascular coagulation and COVID‐associated coagulopathy [19]. Despite early concerns from autopsy series and proposed models of pathogenesis, COVID as a trigger for complement‐mediated TMA is controversial and is limited to case reports [19, 20, 21, 22, 23]. If SARS‐CoV‐2 infection were to trigger endothelial injury in the donor, it is unclear if there would be sustained effects on the allograft in the post‐transplant setting. Calcineurin inhibitors, AMR, and other viral infections, and patient‐specific immunologic risk are known factors associated with TMA in the post‐transplant setting [18, 24, 25]. Thus, the single case of de novo TMA with complement dysregulation 10 days after transplant from a CoV‐COD donor cannot be presumptively ascribed to the donor kidney.

As previously reported, our donor groups based on SARS‐CoV‐2 status were significantly different, with NAT+ donors being younger and less likely to be Caucasian, and COVID‐COD donors being universally DCD, having higher BMI, and longer cold ischemic times. When controlled for variables that also affect allograft outcome, we found no significant difference in 3‐year eGFR based on donor CoV status for recipients of NAT+ and CoV‐COD kidneys. Similar comparative outcomes out to 2 years have now been reported for the larger group of kidney transplants from donors defined as active, resolved, and negative for COVID‐19 identified in the United Network for Organ Sharing (UNOS) database [4]. Yamauchi et al. reported 6‐month outcomes for a smaller, though highly propensity‐matched cohort of kidney transplants from SARS‐CoV‐2 positive donors [25]. They found significantly lower eGFR for their subset of 15 recipients of kidneys from donors who died of COVID‐19 compared to matched recipients of SARS‐CoV‐2‐negative donors. The 1‐year eGFR for CoV‐COD donors in our study appears to be similarly affected, though 3‐year eGFR differences did not meet statistical significance, and the multivariable analysis did not support an effect. Given the retrospective nature of the study and lack of propensity matching, it is possible that such differences are meaningful even without overt pathologic findings to explain them. It is unclear whether such effects would be related to severe COVID itself, its therapies, or to other events during the donor's terminal hospitalization.

As pointed out in our previous report, the donors included a range of SARS‐CoV‐2 infection statuses. One‐third had evidence for active infection by imaging and cycle threshold measures. This is compared to many reports where it appears that decisions to accept organs were based on a low likelihood of active infection. Active infection remains of importance to procurement teams and airborne/droplet precautions remain relevant. However, our data suggests that the presence of active SARS‐CoV‐2 infection in the donor is not specifically relevant to outcomes for kidney recipients. UNOS data for heart recipients from donors loosely defined as having active infection suggests an increased early mortality compared to recipients of organs from NAT‐neg donors or those defined as recently resolved [26]. This implies that there may be organ‐specific differences in outcomes. There may be a reason to better define what constitutes an active infection in a potential organ donor outside of NAT positivity. Where able, it may be of value to assess outcomes for recipients of different extrapulmonary organs from the same SARS‐CoV‐2‐infected donor to assess organ‐specific effects.

Our study is limited by relatively low numbers that may not have allowed small differences to be detected. It is also limited by a lack of propensity matching for baseline characteristics, a potentially important missing factor. Larger multicenter data with similar granular findings are warranted to avoid center‐specific biases. Also, data such as that from Goldman et al. using UNOS data to assess longer‐term allograft and patient survival is awaited [2, 25].

In the most detailed report to date, with 57 mate kidneys included, we found that recipients of kidneys from donors that were NAT+ or CoV‐COD experienced similar 3‐year patient‐related and allograft outcomes to those receiving organs from NAT‐negative donors. Biopsy pathologies and rejection at 1 year were no different and recipients of mate kidneys from the same SARS‐CoV‐affected donors did not experience the same events. Significant subclinical pathologies from SARS‐CoV‐2 infection were not identified in this study, though unmeasured effects on those dying from severe COVID may modestly reduce recipient eGFR.

## Author Contributions

C.K. participated in research design, writing the paper, performing the research, and data analysis. M.E., E.P., and A.W. participated in the research design and performance of the research. N.S. participated in research design, data analysis, and writing of the paper. L.H. participated in the performance of research and writing of the paper.

## Funding

The authors have nothing to report.

## Conflicts of Interest

The authors declare no conflicts of interest.

## Supporting information




**Supporting Table 1:** Kidney transplant (KT) recipients with thrombotic microangiopathy (TMA) on post‐transplant biopsy.

**Supporting File 1**: tid70202‐sup‐0002‐VisualAbstract.pptx

## Data Availability

The data that support the findings of this study are available from the corresponding author upon reasonable request.
