# Supplementary material for: Comparative 3‐Year Allograft Outcomes for Recipients of Kidneys From SARS‐CoV‐2 NAT‐Positive Donors
Source: Transpl Infect Dis. 2026 Mar 13;28(4):e70202. doi: 10.1111/tid.70202 (PMC13450162; doi:10.1111/tid.70202)
Supplement: Supplementary file 1 [file TID-28-e70202-s001.docx]

Supplemental table. Kidney transplant (KT) recipients with thrombotic microangiopathy (TMA) on post-transplant biopsy

|  | Patient 1  NAT+ donor | Patient 2  CoV-COD donor | Patient 3  NAT neg donor |
| --- | --- | --- | --- |
| Underlying disease | Sjogren’s interstitial nephritis with TMA  PD x 3 yr | FSGS  PD x 4yr | Hypertensive nephropathy  HD x 5yr |
| Time transplant to TMA on biopsy | 88d | 10d | 186d |
| Other findings | Recurrent GN  ADAMTS13 normal | ADAMTS13 103  C3, C4 normal  SMAC normal | Prior ACR 1b |
| DSA | Yes | Yes, de novo | No |
| Treatment | eculizumab | eculizumab, PLEX, belatacept | none additional |
| eGFR, 1 year, mL/min/m^2^ | Died 109d post KT  33 | 28  (18 mos: 37) | 21 |
| Donor COVID details | Ct not done  3d 1^st^ +, still +  Normal Xray | Ct not done  16d 1st +, still +  Multifocal infiltrates | NA |
| Donor KDPI  Cr mg/dL | KDPI 59  Cr 0.49 | KDPI 76  Cr 1.08 | KDPI 49  Cr 0.56 |
| Mate Kidney, eGFR, mL/min/m^2^ | Normal biopsy  eGFR 33 | Not allocated | Normal biopsy,  eGFR 21 |

PD, peritoneal dialysis; HD hemodialysis; GN, glomerulonephritis; ADAMTS13, a disintegrin and metalloproteinase with a thrombospondin type 1 motif, member 13; C3, complement component 3; C4 complement component 4; SMAC, soluble membrane attack complex; ACR, acute cellular rejection; PLEX, plasma exchange; Ct, cycle threshold; KDPI kidney donor profile index; eGFR, estimated glomerular filtration rate.
